# Comparing the Maryland Comprehensive Cancer Control Plan With Federal Cancer Prevention and Control Recommendations

**DOI:** 10.5888/pcd12.150008

**Published:** 2015-10-01

**Authors:** Stephanie L. Fowler, Elizabeth A. Platz, Marie Diener-West, Sarah Hokenmaier, Meredith Truss, Courtney Lewis, Norma F. Kanarek

**Affiliations:** Author Affiliations: Elizabeth A. Platz, Department of Epidemiology, Johns Hopkins Bloomberg School of Public Health, and the Sidney Kimmel Comprehensive Cancer Center at Johns Hopkins, Baltimore, Maryland; Marie Diener-West, Department of Biostatistics, Johns Hopkins Bloomberg School of Public Health, Baltimore, Maryland; Sarah Hokenmaier, Meredith Truss, Courtney Lewis, Maryland Department of Health and Mental Hygiene, Center for Cancer Prevention and Control, Baltimore, Maryland; Norma F. Kanarek, Department of Environmental Health Sciences, Johns Hopkins Bloomberg School of Public Health, and the Sidney Kimmel Comprehensive Cancer Center at Johns Hopkins, Baltimore, Maryland.

## Abstract

**Introduction:**

Since the introduction of the Affordable Care Act (ACA) in 2012, 11 million more Americans now have access to preventive services via health care coverage. Several prevention-related recommendations issued by the US Preventive Services Task Force (USPSTF), Centers for Disease Control and Prevention (CDC), and Advisory Committee on Immunization Practices (ACIP) are covered under the ACA. State cancer plans often provide prevention strategies, but whether these strategies correspond to federal evidence-based recommendations is unclear. The objective of this article is to assess whether federal evidence-based recommendations, including those covered under the ACA, are included in the Maryland Comprehensive Cancer Control Plan (MCCCP).

**Methods:**

A total of 19 federal recommendations pertaining to cancer prevention and control were identified. Inclusion of federal cancer-related recommendations by USPSTF, CDC, and ACIP in the MCCCP’s goals, objectives, and strategies was examined.

**Results:**

Nine of the federal recommendations were issued after the MCCCP’s publication. MCCCP recommendations corresponded completely with 4 federal recommendations and corresponded only partially with 3. Reasons for partial correspondence included specification of less restrictive at-risk populations or different intervention implementers. Three federal recommendations were not mentioned in the MCCCP’s goals, objectives, and strategies.

**Conclusion:**

Many cancer-related federal recommendations were released after the MCCCP’s publication and therefore do not appear in the most current version. We recommend that the results of this analysis be considered in the update of the MCCCP. Our findings underscore the need for a periodic scan for changes to federal recommendations and for adjusting state policies and programs to correspond with federal recommendations, as appropriate for Marylanders.

## Introduction

The Centers for Disease Control and Prevention (CDC) funds all 50 states through the National Comprehensive Cancer Control Program (NCCCP) to develop and implement comprehensive cancer control plans (1). There is no standard national cancer plan; instead, state cancer plans vary on the basis of content and organization, depending on a host of factors including state priorities, political climate, and stakeholder involvement and partnership. Furthermore, cancer plans are not static but are updated and revised on an ongoing basis. For example, Maryland revises its plan every 3 to 5 years (2). According to CDC, one goal of comprehensive cancer control planning is to disseminate and implement best practices and evidence-based strategies (1). The US Preventive Services Task Force (USPSTF), CDC, and Advisory Committee on Immunization Practices (ACIP) all issue cancer-related evidence-based recommendations (3–5). USPSTF’s grade A and B recommendations and many of CDC’s and ACIP’s recommendations and guidelines are covered under the Affordable Care Act (ACA) (3–5). To our knowledge, there has not been an assessment of any state cancer plan examining the extent to which these recommendations are included. 

The first Maryland cancer plan was written in 1991 and has been updated several times since. The most recent plan, published in 2011, was developed by more than 200 contributors including representatives from hospitals, academia, nongovernmental organizations, and government, as well as citizens. Contributors formed committees to write specific chapters in the Maryland Comprehensive Cancer Control Plan (MCCCP). The process included an extensive review of evidence-based recommendations from multiple sources that were published when the plan was being developed. Recommendations from reputable sources such as USPSTF, CDC, the American Cancer Society, and many others relevant to the specific topic area were reviewed and considered by the MCCCP contributors. In addition, other recently published Maryland health plans were reviewed to achieve a cohesive approach to the development of goals, objectives, and strategies. MCCCP contributors used these recommendations, Maryland-specific data, and the expertise of many local physicians, academicians, public health practitioners, community health workers, cancer survivors, and others to determine the final goals, objectives, and strategies to be included in the MCCCP.

The 15-chapter plan focuses on primary, secondary, and tertiary cancer prevention and control and includes cross-cutting surveillance and health disparities sections. Priorities for overcoming the issues described in the MCCCP are presented at the end of each chapter in the form of goals, objectives, and strategies (2), all of which are actionable activities that can be implemented by professionals working in the field of cancer prevention and control. 

The purpose of this investigation was to compare the goals, objectives, and strategies in the Maryland Comprehensive Cancer Control Plan (MCCCP) (2) with the cancer-related, evidence-based recommendations of federal agencies that are covered under the ACA. We examined the extent to which cancer-related USPSTF recommendations (grade A and B only), ACIP recommendations, and CDC guidelines were included in the 2011 MCCCP’s goals, objectives, and strategies. The goal of this assessment is to inform future updates of the MCCCP and to provide a model for other state cancer plans.

## Methods

We reviewed recommendations issued by the USPSTF, ACIP, and CDC. The rationale for using these authoritative bodies was their extensive reliance on evidence-based medicine in addition to their cancer-related recommendations covered under the ACA. The Agency for Healthcare Research and Quality (6) and the National Cancer Institute (7) websites refer to USPSTF recommendations, which supports our selection of the USPSTF. USPSTF and ACIP recommendations and CDC guidelines are updated periodically. Nineteen cancer prevention and control-related recommendations or guidelines released by May 31, 2014 were included in our assessment (Table).

**Table:** Correspondence Between 19 Federal Evidence-Based Recommendations and Guidelines^a^ for Primary and Secondary Cancer Prevention and Control and Recommendations in the 2011 Maryland Comprehensive Cancer Control Plan (MCCCP)

MCCCP Published Before Recommendations
Clinicians provide counseling about minimizing exposure to ultraviolet radiation to reduce risk for skin cancer, aged 10 to 24 years with fair skin. (USPSTF Grade B; issued May 2012)
Screening all adults for obesity. Clinicians should offer or refer patients with a body mass index (BMI) of 30 kg/m^2^ or higher to intensive, multicomponent behavioral interventions. (USPSTF Grade B; issued June 2012)
Annual screening for lung cancer with low-dose computed tomography in adults aged 55 to 80 years who have a 30 pack-year smoking history and currently smoke or have quit within the past 15 years. Screening should be discontinued once a person has not smoked for 15 years or develops a health problem that substantially limits life expectancy or the ability or willingness to have curative lung surgery. (USPSTF Grade B; issued December 2013)
Primary care providers screen women who have family members with breast, ovarian, tubal, or peritoneal cancer with one of several screening tools designed to identify a family history that may be associated with an increased risk for potentially harmful mutations in breast cancer susceptibility genes (BRCA1 or BRCA2). Women with positive screening results should receive genetic counseling and, if indicated after counseling, BRCA testing. (USPSTF Grade B; issued December 2013)
Clinicians engage in shared, informed decision-making with women who are at increased risk for breast cancer about medications to reduce their risk. For women who are at increased risk for breast cancer and at low risk for adverse medication effects, clinicians should offer to prescribe risk-reducing medications, such as tamoxifen or raloxifene. (USPSTF Grade B; issued September 2013)
Screening for cervical cancer in women aged 21 to 65 years with cytology (Pap smear) every 3 years or, for women aged 30 to 65 years who want to lengthen the screening interval, screening with a combination of cytology and human papillomavirus (HPV) testing every 5 years. (USPSTF Grade A; issued March 2012)
Screening for hepatitis C virus (HCV) infection in persons at high risk for infection. Recommended one-time screening for HCV infection to adults born between 1945 and 1965. (USPSTF Grade B; issued June 2013)
Screening for hepatitis B virus (HBV) infection in persons at high risk for infection. (USPSTF Grade B; issued May 2014)
Clinicians screen adults aged 18 years or older for alcohol misuse and provide persons engaged in risky or hazardous drinking with brief behavioral counseling interventions to reduce alcohol misuse. (USPSTF Grade B; issued May 2013)
**Complete Correspondence**
Promote and support breastfeeding. (USPSTF Grade B; issued October 2008)
Screening mammography for women, with or without clinical breast examination, every 1 to 2 years for women age 40 years or older. (USPSTF Grade B; issued September, 2002)
Proper levels of physical activity: 2 hours and 30 minutes (150 min) of moderate-intensity aerobic activity (ie, brisk walking) every week and weight training muscle-strengthening activities on 2 or more days a week that work all major muscle groups (legs, hips, back, abdomen, chest, shoulders, and arms). OR 1 Hour and 15 minutes (75 min) of vigorous-intensity aerobic activity (ie, jogging or running) every week and weight training muscle-strengthening activities on 2 or more days per week that work all major muscle groups (legs, hips, back, abdomen, chest, shoulders, and arms). OR An equivalent mix of moderate- and vigorous-intensity aerobic activity and weight training muscle-strengthening activities on 2 or more days per week that work all major muscle groups (legs, hips, back, abdomen, chest, shoulders, and arms). (CDC; issued in 2008)
Routine vaccination of HPV2 and HPV4 of females aged 11 or 12 years, and catch-up vaccination for females aged 13 through 26 years^b^. (ACIP; issued March 2007)
**Partial Correspondence: Less Restrictive At-Risk Population in MCCCP**
Screening for colorectal cancer using fecal occult blood testing (FOBT), sigmoidoscopy, or colonoscopy, in adults, beginning at age 50 years and continuing until age 75 years. (USPSTF Grade A; issued October 2008) MCCCP recommends screening for those 50 or older per ACS/Multi-Society Taskforce guidelines^c^.
**Partial Correspondence: Different Implementer**
Clinicians ask all adults about tobacco use and provide tobacco cessation interventions for those who use tobacco products. (USPSTF Grade A; issued April 2009) MCCCP states hospitals should adopt inpatient counseling and treatment of tobacco-using patients^c^.
Clinicians ask all pregnant women about tobacco use and provide augmented pregnancy-tailored counseling to those who smoke. (USPSTF Grade A; issued April 2009) MCCCP states hospitals should adopt inpatient counseling and treatment of tobacco-using patients^c^.
**No Mention in Goals, Objectives, and Strategies**
Screening for HBV infection in pregnant women at their first prenatal visit. (USPSTF Grade A; issued June 2009)
Intensive behavioral dietary counseling . . . diet-related chronic disease. Intensive counseling can be delivered by primary care clinicians or by referral to other specialists, such as nutritionists or dietitians^d^. (USPSTF Grade B; issued January 2003)
According to the Dietary Guidelines for Americans, moderate alcohol consumption is defined as having up to 1 drink per day for women and up to 2 drinks per day for men^e^. (CDC; issued in 2010)

Abbreviations: ACIP, Advisory Committee on Immunization Practices; ACS, American Cancer Society; CDC, Centers for Disease Control and Prevention; Pap, Papanicolaou; USPSTF, US Preventive Services Task Force.

a Federal primary and secondary cancer prevention and control recommendations and guidelines from the USPSTF (3), CDC (4), and ACIP (5).

b MCCCP does not include specific age range, but refers to “guideline-eligible” populations.

c Explanation for MCCCP classification.

d USPSTF recommendation is specific to people with risk factors for cardiovascular disease.

e Appears in MCCCP text but not in the goals, objectives, and strategies.

For this analysis, we selected federal recommendations or guidelines pertaining to cancer prevention and control for adults. Because tobacco use, physical inactivity, obesity, and alcohol misuse increase the risk of developing cancer (8,9), we included in our analysis recommendations and guidelines pertaining to these behaviors and conditions. We identified 16 USPSTF recommendations (3) concerning breastfeeding, dietary counseling, risk reduction and genetic screening for women at high risk for breast cancer, early cancer detection screening, obesity, tobacco use cessation, diet, alcohol use, sun exposure, and hepatitis B virus (HBV) and hepatitis C virus (HCV) screening. In addition, we identified one ACIP recommendation (5) that did not correspond to the USPSTF recommendation pertaining to human papillomavirus (HPV) vaccination. From CDC guidelines (4), one physical activity and one healthy alcohol use guideline was identified. There was complete overlap between the USPSTF and ACIP recommending screening for HBV infection in pregnant women and in adults at high risk. Both the USPSTF and ACIP recommend that “all pregnant women should be tested routinely for HBsAg during an early prenatal visit” and that “all adults at risk for HBV infection be screened” (HbsAg is an HBV surface antigen). We used the USPSTF recommendations, because they were rated as grade A (testing for surface antigens in pregnant women) and grade B (testing for surface antigens in high-risk adults). Additionally, the USPSTF 2002 (as opposed to the 2009) mammography recommendation was adopted by the ACA and is the one included in this assessment.

If a federal recommendation or guideline was issued after the release of the MCCCP (July 2011), it was classified as “MCCCP published prior to recommendation.” If the MCCCP was released after the federal recommendation was issued, it was classified as “complete correspondence” if the MCCCP met 3 specific criteria: the goal, objective, and strategies had to 1) recommend the same implementer as the federal recommendation or guideline, 2) recommend the same intervention as the federal recommendation or guideline, and 3) direct the implementation to the same target population as the federal recommendation or guideline. If an MCCCP strategy met at least one but not all of these criteria, it was classified as “partial correspondence.” If a federal recommendation or guideline was issued before the 2011 MCCCP but did not appear in the MCCCP’s goals, objectives, and strategies, it was classified as “no mention.”

## Results

We identified 19 federal recommendations and guidelines pertaining to primary and secondary cancer prevention and control for adults (Table). Nine recommendations, all from the USPSTF, were issued after the release of the MCCCP in July 2011 (47.4%). Of the remaining 10, complete correspondence was found for 4 recommendations (21.1%) in the domains of breast feeding, early detection screening, vaccinations, and physical activity. There was partial correspondence for 3 recommendations and guidelines (15.8%). Reasons for partial correspondence stemmed from the MCCCP strategy having less restrictive at-risk populations (eg, screening age for colorectal cancer ending at 75 [USPSTF] vs no age limit [MCCCP], n = 1) and from having specified alternate intervention implementers (eg, physicians [USPSTF] vs hospitals [MCCCP] engaged in smoking cessation, n = 2). Three of the federal recommendations or guidelines were in place at the time of the MCCCP’s publication but were not mentioned in the MCCCP’s goals, objectives, and strategies (15.8%).

## Discussion

We assessed the extent to which selected federal evidence-based recommendations covered under the ACA were included in the current MCCCP. The MCCCP did not include many of the federal recommendations considered by this study or had partial correspondence. The MCCCP is updated every 5 years, so although it is intended to be current and inclusive, the 2011 MCCCP predated the issuance of many of the federal recommendations. Reasons for partial correspondence stemmed from selected federal recommendations differing from other authoritative sources consulted during the development of the MCCCP, such as the American Cancer Society and the American Congress of Obstetricians and Gynecologists. Because the MCCCP predated the introduction of the ACA, the current assessment approach could not have been used to select strategies during the development of the current MCCCP. However, going forward, such an assessment could be used to update the MCCCP and other state cancer plans. Below we discuss how to ensure that recommendations covered by the ACA are included in the updated MCCCP.

### MCCCP published before recommendations

Of the 19 federal recommendations or guidelines identified, 9 were issued after the MCCCP was released in July 2011. USPSTF and ACIP recommendations and CDC guidelines are revised as the scientific evidence base is refined; the timing of those revisions and additions do not necessarily align with the 5-year cycles of the MCCCP. Full revision of the MCCCP is a massive undertaking by hundreds of contributors resulting in a consensus document. Therefore, wholesale revision of the MCCCP each time a recommendation is revised or added may not be feasible. Hence, including links to sources of up-to-date recommendations in the MCCCP and providing access to updated recommendations through addendums released online between the more comprehensive revisions that occur every 5 years is recommended. The Center for Cancer Prevention and Control (CCPC) in the Cancer and Chronic Disease Bureau of the Maryland Department of Health and Mental Hygiene (DHMH) uses this practice to keep the MCCCP current between the 5-year cycles.

### No mention in goals, objectives, and strategies

Three of the national recommendations were in place before July 2011 but were not included in the MCCCP’s goals, objectives, and strategies. The site-specific chapters of the MCCCP focus on 7 cancers that are targeted by the state’s Cigarette Restitution Fund (CRF) Program (breast, cervical, colorectal, lung, melanoma, oral, and prostate), which were selected in 2000 as priorities because of their high burden in the state, modifiable risk factors, or effective early detection methods. One of the 3 recommendations not included addresses HBV, a risk factor for liver cancer (10). Since liver cancer is not one of the CRF-targeted cancers, MCCCP authors decided to address liver cancer only in the disparities chapter and not in its own chapter or in the goals, objectives, and strategies of the current MCCCP. To keep the MCCCP current, it may be advisable to broaden the scope of future versions of the plan to include additional cancer prevention recommendations that address cancer sites beyond the CRF-targeted cancers.

In addition, alcohol misuse, an important modifiable risk factor that contributes to risk of cancer, was not mentioned in the goals, objects, and strategies. The CCPC is “responsible for assessing cancer control in Maryland and developing cancer control priorities through collaboration with the State Council on Cancer Control and other state agencies” (11) and develops and periodically updates the MCCCP. Although other arms within the DHMH coordinate programs and implement primary and secondary cancer prevention and control-related interventions (eg, DHMH Infectious Disease Bureau), alcohol misuse is handled by the Alcohol and Drug Abuse Administration, an arm of the DHMH that did not contribute to the 2011 MCCCP. As a means of facilitating the use of the assessment used in current study for updating future cancer plans, participation from this and other relevant DHMH administrations and centers is recommended.

### Partial correspondence between the MCCCP and national recommendations and guidelines

Three of the federal recommendations and guidelines were included in the MCCCP, but they differed on either the implementer or the targeted population.

**Different implementer.** Two recommendations and guidelines had partial correspondence because the recommended implementer of the interventions differed. Specifically, in 2 cases the MCCCP identified the hospital as the implementer whereas the federal recommendation specified the physician as the implementer of the intervention. Although physician groups did participate in the development of the MCCCP, this highlights the importance of soliciting a more direct and central role in the writing and implementation of the plan’s goals, objectives, and strategies.

**Less restrictive at-risk population in the MCCCP.** Another reason for partial correspondence occurred with the specifics of screening recommendations, primarily the relevant age groups for screening. Although our assessment was limited to USPSTF and CDC recommendations and CDC guidelines, the MCCCP refers to additional national recommendations throughout, including screening recommendations set by the American Cancer Society, the American College of Gastroenterology, the American Dental Association, and the American Congress of Obstetricians and Gynecologists. In addition, the Maryland DHMH convenes state medical advisory groups to address various screening guidelines, including those provided by USPSTF; these recommendations are used in the implementation of the Cancer Prevention, Education, Screening and Treatment Program for the uninsured and underinsured. It may be possible to harmonize payment guidelines with the ACA and MCCCP recommendations. In light of additional national health quality and cost guidelines and life expectancy calculations (12,13), revisiting these recommendations may also be in order.

This assessment assumed that all current cancer-related USPSTF, ACIP, and CDC evidence-based recommendations should be included in state comprehensive cancer control plans. Comparison of the MCCCP’s goals, objectives, and strategies with the selected federal recommendations highlighted the degree of correspondence with the MCCCP, which is the most comprehensive and inclusive cancer plan to date for Maryland. Of the 5 state plans published since 1991, this plan had the most contributors, had the greatest sector representation, and addressed individual, system, and environment concerns. To help maximize health care coverage and access to preventive services, we recommend using this assessment as part of the planning process for future updates of the MCCCP. Incorporating up-to-date evidence-based recommendations, in particular those covered by the ACA, into MCCCP goals, objectives, and strategies is crucial for encouraging state cancer plan users to close the gaps in coverage of these important services. The first step in enabling plan users to implement evidence-based objectives and strategies proven effective at reducing the incidence and burden of cancer is to first incorporate them into the plan. Therefore, comprehensive cancer control plans should be evidence-based to help guide development of statewide priorities that are reflective of the state’s burden to inform implementation strategies and to evaluate impact. In addition to providing important cancer plan funding, another role of CDC in keeping plans up to date would be to support state research priorities through its partnerships with academic institutions and care delivery organizations as a means of informing evolving priorities and generating new evidence.

Updates of the MCCCP may also benefit from the ongoing consideration of recommendations from the Task Force on Community Preventive Services (14), which provides evidence-based recommendations for community-level activities and behaviors, and the expert opinion-based National Comprehensive Cancer Network (15). The main impetus for including the current set of criteria in our assessment was that these interventions are covered under the ACA and would, thus, enhance health (16) and increase access to preventive services for virtually all Marylanders. Other states could assess their plans in a similar manner to the methods described here to optimize population uptake and availability of preventive services (17) that are both evidence-based and cost saving (18).

## References

[1] Centers for Disease Control and Prevention. National Comprehensive Cancer Control Program; 2013. http://www.cdc.gov/cancer/ncccp/about.htm. Accessed November 26, 2013.

[2] Maryland Comprehensive Cancer Control Plan. 2011. http://phpa.dhmh.maryland.gov/cancer/cancerplan/plan2011/CoverTOCAcknowPreface.pdf. Published July 2011. Accessed November, 25, 2014.

[3] United States Preventive Services Task Force. USPSTF A and B recommendations; 2014. http://www.uspreventiveservicestaskforce.org/Page/Name/uspstf-a-and-b-recommendations. Accessed October 28, 2014.

[4] Centers for Disease Control and Prevention. Recommendations, best practices, and guidelines. http://www.cdc.gov/chronicdisease/resources/guidelines.htm. Accessed November 23, 2014.

[5] Advisory Committee on Immunization Practices. ACIP vaccine recommendations. http://www.cdc.gov/vaccines/hcp/acip-recs/index.html. Updated March 2014. Accessed November 10, 2014.

[6] Agency for Healthcare Research and Quality. US Preventive Services Task Force: an introduction. http://www.ahrq.gov/professionals/clinicians-providers/guidelines-recommendations/uspstf/index.html. Accessed November 20, 2014.

[7] National Cancer Institute. Cancer Control PLANET. http://cancercontrolplanet.cancer.gov/index.html. Accessed November 20, 2014.

[8] Colditz GA , DeJong D , Hunter D , Trichopolous D , Willett W . Harvard report on cancer prevention (vol. 1): causes of human cancer. Can Causes Control 1996;7:1–59.

[9] Colditz GA , Samplin-Salgado M , Ryan CT , Dart H , Fisher L , Tokuda A , ; Harvard Center for Cancer Prevention. Harvard report on cancer prevention, volume 5: fulfilling the potential for cancer prevention: policy approaches. Cancer Causes Control 2002;13(3):199–212. 10.1023/A:1015040702565 12020101

[10] Raza SA , Clifford GM , Franceschi S . Worldwide variation in the relative importance of hepatitis B and hepatitis C viruses in hepatocellular carcinoma: a systematic review. Br J Cancer 2007;96(7):1127–34. 10.1038/sj.bjc.6603649 17406349 PMC2360117

[11] Department of Health and Mental Hygiene. Center for Cancer Prevention and Control. http://phpa.dhmh.maryland.gov/cancer/SitePages/Home.aspx. Accessed November 1, 2014.

[12] Hoffman RM , Walter LC . Colorectal cancer screening in the elderly: the need for informed decision making. J Gen Intern Med 2009;24(12):1336–7. 10.1007/s11606-009-1121-7 19798537 PMC2787939

[13] Lantz PM , Ubel PA . The use of life expectancy in cancer screening guidelines. Moving with caution from model-based evidence to evidence-based guidelines. J Gen Intern Med 2005;20(6):552–3. 10.1111/j.1525-1497.2005.41012.x 15987335 PMC1490128

[14] Community Preventive Services Task Force. http://www.thecommunityguide.org/about/conclusionreport.html. Accessed April 25, 2014.

[15] National Comprehensive Cancer Network. NCCN guidelines. http://www.nccn.org/professionals/physician_gls/f_guidelines.asp#detection. Accessed June 3, 2014.

[16] Courtemanche CJ , Zapata D . Does universal coverage improve health? The Massachusetts experience. J Policy Anal Manage 2014;33(1):36–69. 10.1002/pam.21737 24358528

[17] Abed J , Reilley B , Butler MO , Kean T , Wong F , Hohman K . Developing a framework for comprehensive cancer prevention and control in the United States: an initiative of the Centers for Disease Control and Prevention. J Public Health Manag Pract 2000;6(2):67–78. 10.1097/00124784-200006020-00011 10787781

[18] Olchanski N , Cohen JT , Neumann PJ . A role for research: an observation on preventive services for women. Am J Prev Med 2013;44(1, Suppl 1):S12–5. 10.1016/j.amepre.2012.09.016 23195158

